# Identifying Disease-Gene Associations With Graph-Regularized Manifold Learning

**DOI:** 10.3389/fgene.2019.00270

**Published:** 2019-04-02

**Authors:** Ping Luo, Qianghua Xiao, Pi-Jing Wei, Bo Liao, Fang-Xiang Wu

**Affiliations:** ^1^Division of Biomedical Engineering, University of Saskatchewan Saskatoon, SK Canada; ^2^School of Mathematics and Physics, University of South China, Hengyang, China; ^3^College of Computer Science and Technology, Anhui University, Hefei, China; ^4^School of Mathematics and Statistics, Hainan Normal University, Haikou, China; ^5^Department of Mechanical Engineering, University of Saskatchewan, Saskatoon, SK, Canada; ^6^Department of Computer Science, University of Saskatchewan, Saskatoon, SK, Canada

**Keywords:** disease gene identification, manifold learning, disease module theory, gene ontology, multi-task learning

## Abstract

Complex diseases are known to be associated with disease genes. Uncovering disease-gene associations is critical for diagnosis, treatment, and prevention of diseases. Computational algorithms which effectively predict candidate disease-gene associations prior to experimental proof can greatly reduce the associated cost and time. Most existing methods are disease-specific which can only predict genes associated with a specific disease at a time. Similarities among diseases are not used during the prediction. Meanwhile, most methods predict new disease genes based on known associations, making them unable to predict disease genes for diseases without known associated genes.In this study, a manifold learning-based method is proposed for predicting disease-gene associations by assuming that the geodesic distance between any disease and its associated genes should be shorter than that of other non-associated disease-gene pairs. The model maps the diseases and genes into a lower dimensional manifold based on the known disease-gene associations, disease similarity and gene similarity to predict new associations in terms of the geodesic distance between disease-gene pairs. In the 3-fold cross-validation experiments, our method achieves scores of 0.882 and 0.854 in terms of the area under of the receiver operating characteristic (ROC) curve (AUC) for diseases with more than one known associated genes and diseases with only one known associated gene, respectively. Further *de novo* studies on Lung Cancer and Bladder Cancer also show that our model is capable of identifying new disease-gene associations.

## 1. Introduction

Complex diseases are caused by a group of genes known as disease genes. Identifying disease-gene associations is of critical importance since it helps us unravel the mechanisms of diseases, which has many applications such as diagnosis, treatment and prevention of disease. With the advances in high-throughput experimental techniques, a large amount of data that indicate associations between diseases and their associated genes have been generated, which could accelerate the identification of disease-associated genes. However, it is expensive and time-consuming to experimentally prove an association between a gene and a disease. Computational methods that translate the experimental data into legible disease-gene associations are necessary for in-depth experimental validation.

Currently, many algorithms have been developed to predict disease-gene associations, and they can be briefly divided into two categories: the machine learning-based methods and the network-based methods. The typical machine learning-based methods extract gene-related features and train models that can discriminate disease genes and passenger genes (Mordelet and Vert, 2011; Yang et al., 2012; Singh-Blom et al., 2013; Luo et al., 2019a,b). Since the features are extracted for genes, these algorithms are usually single-task algorithms which once can only predict disease genes for a specific disease. Thus, for diseases that have a few or no known associated genes, the number of the genes would be too small to train the model. In the meantime, the relationships among diseases are usually not used in the prediction since only one disease is considered at a time. Matrix completion methods, as a type of machine learning methods, can solve the above two issues by jointly predicting disease-gene associations and leveraging the similarities among diseases during the calculation (Natarajan and Dhillon, 2014; Zeng et al., 2017). However, matrix completion methods generally do not have the global optimal solutions and could take a very long time to converge to even a local optimal solution. Network-based methods are based on the assumption that genes close related in the network are associated with the same diseases. Centrality indices, random walk and network energy are used in many methods to predict disease-gene associations (Köhler et al., 2008; Vanunu et al., 2010; Chen et al., 2014a,b). Although most network-based methods are not affected by the above two issues, their performance is strongly affected by the quality of networks, and they usually perform worse than machine learning-based methods on diseases with many known associated genes (Chen et al., 2015, 2016).

In this study, we propose a manifold learning-based method (dgManifold) to predict disease-gene associations. In our dgManifold, genes and diseases are regarded as points in the same high-dimensional Euclidean space. Our assumption is that diseases and their associated genes should be consistent in some lower dimensional manifold, and the geodesic distance between a disease and its associated genes should be shorter than that of other non-associated disease-gene pairs. Although the Euclidean distance between diseases and genes in the high-dimensional space may not reflect their true geodesic distance, we can map the diseases and genes into a low-dimensional manifold based on the experimentally verified disease-gene associations (Tenenbaum et al., 2000; Ham et al., 2005). Then, the true geodesic distance between all the disease-gene pairs can be calculated. In the meantime, the mapping process is regularized by two affinity graphs, disease similarity network and gene similarity network, so that the learned representations with the similarity information can further increase the prediction accuracy. Additionally, since our dgManifold is a supervised method, and it is difficult (if possible) to learn valuable representations for diseases that only have a few or no known associated genes. A prior information vector calculated with the disease similarities and known disease-gene associations should be combined with the original association data to solve this issue. Similar strategies have been applied to calculate the initial probabilities used in the random walk, which have improved the accuracy of predicting miRNA-disease associations (Chen et al., 2016b, 2018a,b).

In the rest of the manuscript, section 2 describes our algorithm as well as the data sources and evaluation metrics used in the study. Section 3 discusses the evaluation results. Section 4 draws some conclusions.

## 2. Materials and Methods

### 2.1. General Model

Given *n* diseases and *m* genes, the associations among them can be represented by a matrix *A*∈*R*^*n*×*m*^ in which *a*_*ij*_ = 1 if disease *i* is associated with gene *j*, and otherwise *a*_*ij*_ = 0. Intuitively, each disease can be represented by a binary m-dimensional row vector while each gene can be represented by a binary n-dimensional column vector. However, in these high-dimensional spaces, it is hard to calculate the actual distance between a disease and a gene.

If we map the diseases and genes into the same manifold with a lower dimensionality and assume that the distance between a disease and its associated genes should be as short as possible on this manifold, predicting disease-gene associations can be solved by computing this mapping based on known disease-gene associations, which can be mathematically formulated as: finding *k*-dimensional representatives of diseases **r**_1_, …, **r**_*n*_ and *k*-dimensional representatives of genes **q**_1_, …, **q**_*m*_ such that the following objective function is minimized

(1) $$\begin{array}{r} O_{k}=\overset{n}{\underset{i=1}{\sum }}\overset{m}{\underset{j=1}{\sum }}a_{ij}||r_{i}-q_{j}|{|}^{2}. \end{array}$$

However, without any constraints, the objective function (1) is not well defined. To illustrate this, if *k*-dimensional vectors ${\text{r}}_{i}^{+}$ and ${\text{q}}_{j}^{+}$ for *i* = 1, …, *n* and *j* = 1, …, *m* minimize the objective function (1), then $\epsilon {\text{r}}_{i}^{+}$ and $\epsilon {\text{q}}_{j}^{+}$ can further minimize the objective function when 0 ≤ ϵ < 1. Especially, when ϵ = 0, any *k*-dimensional vectors ${\text{r}}_{i}^{+}$ and ${\text{q}}_{j}^{+}$ can minimize the objective function. Therefore, to make the optimization problem well defined, the following constraints are added

(2) $$\begin{array}{r} \overset{n}{\underset{i=1}{\sum }}r_{i}r_{i}^{T}=I_{k}\;\text{and}\;\overset{m}{\underset{j=1}{\sum }}q_{j}q_{j}^{T}=I_{k}. \end{array}$$

where *I*_*k*_ is the *k*×*k* identity matrix. As a results, the learned representations are unique with these constraints.

To insure that the mapped representations of diseases and genes are in concert with their intrinsic properties, two affinity graphs, disease similarity network and gene similarity network are used to regularize the objective function (1), and the new objective function is as follows

(3) $$\begin{array}{r} O_{k}=\overset{m}{\underset{j=1}{\sum }}\overset{n}{\underset{i=1}{\sum }}a_{ij}||r_{i}-q_{j}|{|}^{2}+\frac{\alpha }{2}\overset{n}{\underset{i=1}{\sum }}\overset{n}{\underset{j=1}{\sum }}s_{ij}^{d}||r_{i}-r_{j}|{|}^{2}\\ +\frac{\beta }{2}\overset{m}{\underset{i=1}{\sum }}\overset{m}{\underset{j=1}{\sum }}s_{ij}^{g}||q_{i}-q_{j}|{|}^{2} \end{array}$$

where *S*^*d*^ and *S*^*g*^ are the adjacency matrices of the disease similarity network and the gene similarity network, respectively.

Note that

(4) $$\begin{array}{r} \begin{array}{c} O_{k}=\overset{n}{\underset{i=1}{\sum }}\left(\overset{m}{\underset{j=1}{\sum }}a_{ij}\right)r_{i}^{T}r_{i}+\overset{m}{\underset{j=1}{\sum }}\left(\overset{n}{\underset{i=1}{\sum }}a_{ij}\right)q_{j}^{T}q_{j}-2\overset{n}{\underset{i=1}{\sum }}\overset{m}{\underset{j=1}{\sum }}a_{ij}r_{i}^{T}q_{j}\\ +\alpha \overset{n}{\underset{i=1}{\sum }}\left(\overset{n}{\underset{j=1}{\sum }}s_{ij}^{d}\right)r_{i}^{T}r_{i}-\alpha \overset{n}{\underset{i=1}{\sum }}\overset{n}{\underset{j=1}{\sum }}s_{ij}^{d}r_{i}^{T}r_{j}\\ +\beta \overset{m}{\underset{i=1}{\sum }}\left(\overset{m}{\underset{j=1}{\sum }}s_{ij}^{g}\right)q_{i}^{T}q_{i}-\beta \overset{m}{\underset{i=1}{\sum }}\overset{m}{\underset{j=1}{\sum }}s_{ij}^{g}q_{i}^{T}q_{j}\\ =\overset{n}{\underset{i=1}{\sum }}A_{ri}r_{i}^{T}r_{i}+\overset{m}{\underset{j=1}{\sum }}A_{cj}q_{j}^{T}q_{j}-2\overset{n}{\underset{i=1}{\sum }}\overset{m}{\underset{j=1}{\sum }}a_{ij}r_{i}^{T}q_{j}\\ +\alpha \overset{n}{\underset{i=1}{\sum }}S_{i}^{d}r_{i}^{T}r_{i}-\alpha \overset{n}{\underset{i=1}{\sum }}\overset{n}{\underset{j=1}{\sum }}s_{ij}^{d}r_{i}^{T}r_{j}\\ +\beta \overset{m}{\underset{j=1}{\sum }}S_{j}^{g}q_{j}^{T}q_{j}-\beta \overset{m}{\underset{j=1}{\sum }}\overset{m}{\underset{i=1}{\sum }}s_{ij}^{g}q_{i}^{T}q_{j}\\ =\overset{n}{\underset{i=1}{\sum }}\left(A_{ri}+\alpha S_{i}^{d}\right)r_{i}^{T}r_{i}+\overset{m}{\underset{j=1}{\sum }}\left(A_{cj}+\beta S_{j}^{d}\right)q_{j}^{T}q_{j}\\ -2\overset{n}{\underset{i=1}{\sum }}\overset{m}{\underset{j=1}{\sum }}a_{ij}r_{i}^{T}q_{j}-\alpha \overset{n}{\underset{i=1}{\sum }}\overset{n}{\underset{j=1}{\sum }}s_{ij}^{d}r_{i}^{T}r_{j}-\beta \overset{m}{\underset{j=1}{\sum }}\overset{m}{\underset{i=1}{\sum }}s_{ij}^{g}q_{i}^{T}q_{j} \end{array} \end{array}$$

where $S_{i}^{d}=\overset{n}{\underset{j=1}{\sum }}s_{ij}^{d},S_{i}^{g}=\overset{m}{\underset{j=1}{\sum }}s_{ij}^{g},A_{ri}=\overset{m}{\underset{j=1}{\sum }}a_{ij},A_{cj}=\overset{n}{\underset{i=1}{\sum }}a_{ij}$. Let

(5) $$\begin{array}{r} \begin{array}{c} L^{11}=diag\left[A_{r1}+\alpha S_{1}^{d},A_{r2}+\alpha S_{2}^{d},\ldots ,A_{rn}+\alpha S_{n}^{d}\right]-\alpha S^{d},\\ L^{22}=diag\left[A_{c1}+\beta S_{1}^{g},A_{c2}+\beta S_{2}^{g},\ldots ,A_{cm}+\beta S_{m}^{g}\right]-\beta S^{g}, \end{array} \end{array}$$

the objective function (3) can be simplified as

(6) $$\begin{array}{r} O_{k}=\overset{n}{\underset{i=1}{\sum }}\overset{n}{\underset{j=1}{\sum }}L^{11}r_{i}^{T}r_{j}+\overset{m}{\underset{i=1}{\sum }}\overset{m}{\underset{j=1}{\sum }}L^{22}q_{i}^{T}q_{j}-2\overset{n}{\underset{i=1}{\sum }}\overset{m}{\underset{j=1}{\sum }}a_{ij}r_{i}^{T}q_{j} \end{array}$$

Furthermore, let

(7) $$\begin{array}{r} r_{i}=\left[\begin{array}{c} x_{i1}\\ x_{i2}\\ \vdots \\ x_{ik} \end{array}\right],q_{j}=\left[\begin{array}{c} y_{j1}\\ y_{j2}\\ \vdots \\ y_{jk} \end{array}\right],z_{t}=\left[\begin{array}{c} x_{1t}\\ \vdots \\ x_{nt}\\ y_{1t}\\ \vdots \\ y_{mt} \end{array}\right]=\left[\begin{array}{c} x_{t}\\ y_{t} \end{array}\right], \end{array}$$

(8) $$\begin{array}{r} \begin{array}{c} A_{r}=diag\left[A_{r1},\ldots ,A_{rn}\right],\;A_{c}=diag\left[A_{c1},\ldots ,A_{cm}\right],\\ L^{d}=diag\left[S_{1}^{d},\ldots ,S_{n}^{d}\right]-S^{d},\;L^{g}=diag\left[S_{1}^{g},\ldots ,S_{m}^{g}\right]-S^{g}, \end{array} \end{array}$$

(9) $$\begin{array}{r} L=\left[\begin{array}{c} A_{r}+\alpha L^{d}-A\\ -A^{T}A_{c}+\beta L^{g} \end{array}\right], \end{array}$$

objective function (6) can be simplified as

(10) $$\begin{array}{r} \begin{array}{c} O_{k}=\overset{k}{\underset{t=1}{\sum }}\overset{n}{\underset{i=1}{\sum }}\overset{n}{\underset{j=1}{\sum }}L^{11}x_{it}x_{jt}+\overset{k}{\underset{t=1}{\sum }}\overset{m}{\underset{i=1}{\sum }}\overset{m}{\underset{j=1}{\sum }}L^{22}y_{it}y_{jt}\\ -2\overset{k}{\underset{t=1}{\sum }}\overset{n}{\underset{i=1}{\sum }}\overset{m}{\underset{j=1}{\sum }}a_{ij}x_{it}y_{jt}\\ =\overset{k}{\underset{t=1}{\sum }}\left[x_{t}^{T}L^{11}x_{t}+y_{t}^{T}L^{22}y_{t}-2x_{t}^{T}Ay_{t}\right]\\ =\overset{k}{\underset{t=1}{\sum }}\left[x_{t}^{T}y_{t}^{T}\right]\left[\begin{array}{cc} L^{11}-A\\ -A^{T} & L^{22} \end{array}\right]\left[\begin{array}{c} x_{t}\\ y_{t} \end{array}\right]\\ =Tr\left(Z^{T}LZ\right) \end{array} \end{array}$$

Therefore, minimizing the objective function (4) with constraints (2) is equivalent to minimize the following function

(11) $$\begin{array}{r} Q_{k}=Tr\left(Z^{T}LZ\right) \end{array}$$

with constraints

(12) $$\begin{array}{r} Z^{T}Z=X^{T}X+Y^{T}Y=2I_{k} \end{array}$$

According to Bolla (2013), minimizing objective function (11) with constraints (12) can be solved by

(13) $$\begin{array}{r} Z^{*}=\left(u_{0},u_{1},\ldots ,u_{k-1}\right) \end{array}$$

where **u**_0_, **u**_1_, …, **u**_*k*−1_ are *k* eigenvectors correspond to the *k* smallest eigenvalues of *L*. Meanwhile, the smallest eigenvalue is 0, and the corresponding eigenvector **u**_0_ is a constant vector which does not contribute to the calculation of the geodesic distance. Thus, let Ẑ denote the matrix by removing the fist column of Z^*^. The first *n* rows of Ẑ are the obtained (*k*−1)-dimensional representations of diseases, and the rest *m* rows of Ẑ are the learned representations of genes. The geodesic distance between a disease *i* and gene *j* can be calculated by

(14) $$\begin{array}{r} gdist_{ij}=||{\hat{r}}_{i}-{\hat{q}}_{j}|{|}^{2}. \end{array}$$

### 2.2. Similarity Network

#### 2.2.1. Gene Similarity

In this study, the learning process is regularized by similarity networks, and the similarities of genes are calculated based on the Gene Ontology (GO). GO database provides a set of vocabularies to describe the function of genes and gene products (Ashburner et al., 2000; Consortium, 2017). The GO terms and their relationships are manifested as a directed acyclic graph (DAG) where nodes represent terms while edges represent semantic relationships. Many algorithms have been proposed to calculate the similarities of genes using ontology data, and the approach proposed by Wang et al. (2007) is used in this study.

Let *DAG*_*h*_ = (*T*_*h*_, *E*_*h*_) denote GO term *h*, where *T*_*h*_ contains all the successor GO terms of *h* in the DAG, and *E*_*h*_ contains the semantic relationships between *h* and other terms in *T*_*h*_. Each term *t* in *T*_*h*_ has a τ-value related to *h*:

(15) $$\begin{array}{l} \{\begin{array}{c} \tau_{h}\left(t\right)=1,if\;t=h\\ \tau_{h}\left(t\right)=max\left\{w_{e}*\tau_{h}\left(t^{{}^{\prime }}\right)|t^{{}^{\prime }}\in children\;of\;t\right\},otherwise \end{array} \end{array}$$

where *w*_*e*_ is the weight of the edge (semantic relationships) in the DAG. Two types of semantic relationships (“*is_a*” and “*part_of* ”) are used in the DAG, and the corresponding *w*_*e*_ is set to 0.8 and 0.6, respectively, as recommended in Wang et al. (2007).

Given *DAG*_*h*_ = (*T*_*h*_, *E*_*h*_) and *DAG*_*b*_ = (*T*_*b*_, *E*_*b*_) for GO terms *h* and *b*, their similarity can be computed by

(16) $$\begin{array}{r} sgo\left(h,b\right)=\frac{\sum_{t\in T_{h}\cap T_{b}}\left(\tau_{h}\left(t\right)+\tau_{b}\left(t\right)\right)}{\sum_{t\in T_{h}}\tau_{h}\left(t\right)+\sum_{t\in T_{b}}\tau_{b}\left(t\right)} \end{array}$$

Then, the similarity of one GO term *t*′ and a set of GO terms *GO* = {*t*_1_, *t*_2_, …, *t*_*l*_} is defined as

(17) $$\begin{array}{r} SGO\left(t^{\prime },GO\right)=\max_{1\leq i\leq l}\left(SGO\left(t^{\prime },t_{i}\right)\right) \end{array}$$

Finally, the functional similarity of two genes *g*_1_ and *g*_2_ is calculated by

(18) $$\begin{array}{r} s_{g_{1},g_{2}}^{g}=\frac{\sum_{1\leq i\leq n_{1}}SGO\left(t_{1i},GO_{2}\right)+\sum_{1\leq j\leq n_{2}}SGO\left(t_{2j},GO_{1}\right)}{n_{1}+n_{2}} \end{array}$$

where *GO*_1_ = {*t*_11_, *t*_12_, …, *t*_1_*n*__1__} and *GO*_2_ = {*t*_21_, *t*_22_, …, *t*_2_*n*__2__} are two sets of GO terms that describe *g*_1_ and *g*_2_, respectively.

#### 2.2.2. Disease Similarity

The similarities among diseases are also calculated with the ontology data. Instead of GO, the Human Phenotype Ontology (HPO) (Köhler et al., 2018) is used to characterize human diseases. The HPO provides a vocabulary of phenotypic terms related to human diseases. Each term represents a clinical abnormality, and all the terms are structured as a DAG, in which every term is related to their parent terms by “*is_a*” relationships. Although diseases are not directly described by the HPO, the annotation file provided by HPO contains terms associated with every disease, and thus Equations (17) and (18) can be used to compute the similarities of diseases. When we calculate the similarities of phenotypic terms based on the DAG, *w*_*e*_ in Equation (15) is set to 0.7 as recommended in Li et al. (2011).

### 2.3. Prior Information

For diseases with only a few associated genes, the limited information would affect the performance of any computational algorithms. This problem is especially serious for diseases with no known associated genes. To solve this problem, we add some prior information for diseases with no known associations.

Specifically, given a disease *i*′, ${\text{p}}_{i^{\prime }}$ is added to the *i*′-th row of the matrix *A* as prior information so that the shortage of known information can be alleviated. The *j*-th entry of ${\text{p}}_{i^{\prime }}$ is calculated by

(19) $$\begin{array}{r} p_{i^{\prime }j}=(\overset{n}{\underset{i=1,i\neq i^{\prime }}{\sum }}s_{ii^{\prime }}^{d}a_{ij})/(\overset{n}{\underset{i=1,i\neq i^{\prime }}{\sum }}a_{ij}) \end{array}$$

In our experiments, when cross-validation is used to evaluate the algorithm, the prior information is added to the *i*-th row of matrix *A* as long as one of the associated genes of disease *i* is left to test the model. Meanwhile, in the *de novo* study, prior information is also added to the diseases used for evaluation.

### 2.4. Data Sources

The disease-gene association data are downloaded from the Online Mendelian Inheritance in Man (OMIM) database (Amberger et al., 2014) in August 2018. The Morbid Map at OMIM contains nearly seventy-five hundred entries sorted alphabetically by disorder names. Each entry represents an association between a gene and a disease. Different entries are labeled with different tags (“(3),” “[],” and “?”) which indicate their reliabilities. To obtain a reliable association dataset, based on (Goh et al., 2007), three steps were performed to preprocess the originally downloaded data. First, entries with the tag “(3)” are selected while others are abandoned. We adopt this strategy because diseases with tag “(3)” indicate that the molecular basis of these diseases is known and the associations are reliable, while entries with “[]” represent abnormal laboratory test values, and entries with “?” represent provisional disease-gene associations. Second, disease entries are classified into distinct diseases by merging disease subtypes based on their given disorder names. For instance, 17 entries of “Leigh syndrome” are merged into disease “Leigh syndrome,” and the 19 complementary terms of “Lung cancer somatic” are merged into “Lung Cancer.” Third, 74 diseases are removed because they are not annotated by any HPO terms. During the classification, string match was used to classify adjacent entries, followed by a manual verification. Finally, we obtain a dataset consisting of 4,770 associations between 1,537 diseases and 3,320 genes. Among the 1,537 diseases, 917 have only one associated gene (single-gene disease), while the rest diseases have at least two associated genes (multiple-gene disease).

The ontology data of genes and phenotypes are downloaded from the GO database (Ashburner et al., 2000; Consortium, 2017), and the HPO database (Köhler et al., 2018), respectively. The PPI network used in the competing algorithms is downloaded from the InWeb_InBioMap database (version 2016_09_12) (Li et al., 2016).

### 2.5. Evaluation Metrics

In this study, the algorithm is evaluated in two steps. In the first step, our dgManifold is compared with two competing algorithms: PCFM (Zeng et al., 2017) and Katz (Singh-Blom et al., 2013). PCFM is a matrix completion method which integrates disease similarities and gene similarities to predict disease-gene associations. Katz is a classic network-based method which uses Katz centrality to rank the disease-gene associations. We choose these two algorithms because they are all multi-task algorithms which can predict all disease-gene associations as our dgManifold does. The AUC (area under of the receiver operating characteristic (ROC) curve) scores calculated from 3-fold cross-validation are used to compare these three algorithms.

ROC curve plots the true positive rate [TP/(TP+FN)] verses the false positive rate [FP/(FP+TN)] at different thresholds, and a larger AUC represents better overall performance. In this study, a true positive (TP) is a known disease-gene association (positive sample) predicted as a disease-gene association, while a false positive (FP) is a non-disease-gene association (negative sample) predicted as a disease-gene association. A false negative (FN) is a positive sample predicted as negative while a true negative (TN) is a negative sample predicted as negative. Since negative samples are not included in existing databases, we randomly select a set of unknown disease-gene pairs as negative samples. The number of negative samples is equal to that of positive samples. Considering that the selected negative samples may have small possibilities to be a real disease-gene association, the random selection was run for five times to generate 5 sets of negative samples. The final AUC score is the average score obtained from the 5 sets of samples.

During the cross-validation, the known disease-gene associations are split into 3 groups, and the algorithm is run for 3 rounds. In each round, one group of associations is regarded as unknown (*a*_*ij*_ = 0), while the rest two groups of associations are used to train the model. The prior information is recomputed during every round of the cross-validation. Considering that single-gene diseases would have no known associated genes if they are left for testing the model during the cross-validation, predicting disease genes for these diseases is similar to predict disease genes for a completely new disease. Thus, the three algorithms are compared on multiple-gene diseases and single-gene diseases separately. Additionally, to show the effect of the prior information, the AUC scores of our method without prior information are also calculated.

In the second step, the model is trained with all the known associations, and the geodesic distance between every unknown disease-gene pairs is calculated. To find out whether our new predictions are in concert with existing experimental studies, the top-10 predictions of two diseases, Lung Cancer and Bladder Cancer, are searched from the existing literature. In our dataset, Lung Cancer has 16 associated genes, and Bladder Cancer has 4 associated genes. We choose these two types of cancer because they are experimentally well studied which could better prove our results.

## 3. Results

### 3.1. Model Parameters

In our study, several parameters affect the performance of the model. To obtain the optimal parameters, the grid search is conducted by searching *k* from {20, 30, 50, 100, 500, 800, 1, 000, 1, 200, 1, 500} and α from {0, 0.01, 0.02, 0.05, 0.1, 0.2, 0.5}. β is set to be equal to α. The AUC score is used to determine whether the selected parameters are optimal. Finally, for multiple-gene diseases, the model performs best when *k* = 30, α = β = 0.2, and for single-gene diseases, the optimal parameters are *k* = 30, α = β = 0.1.

### 3.2. Cross-Validation

**Figure 1:**
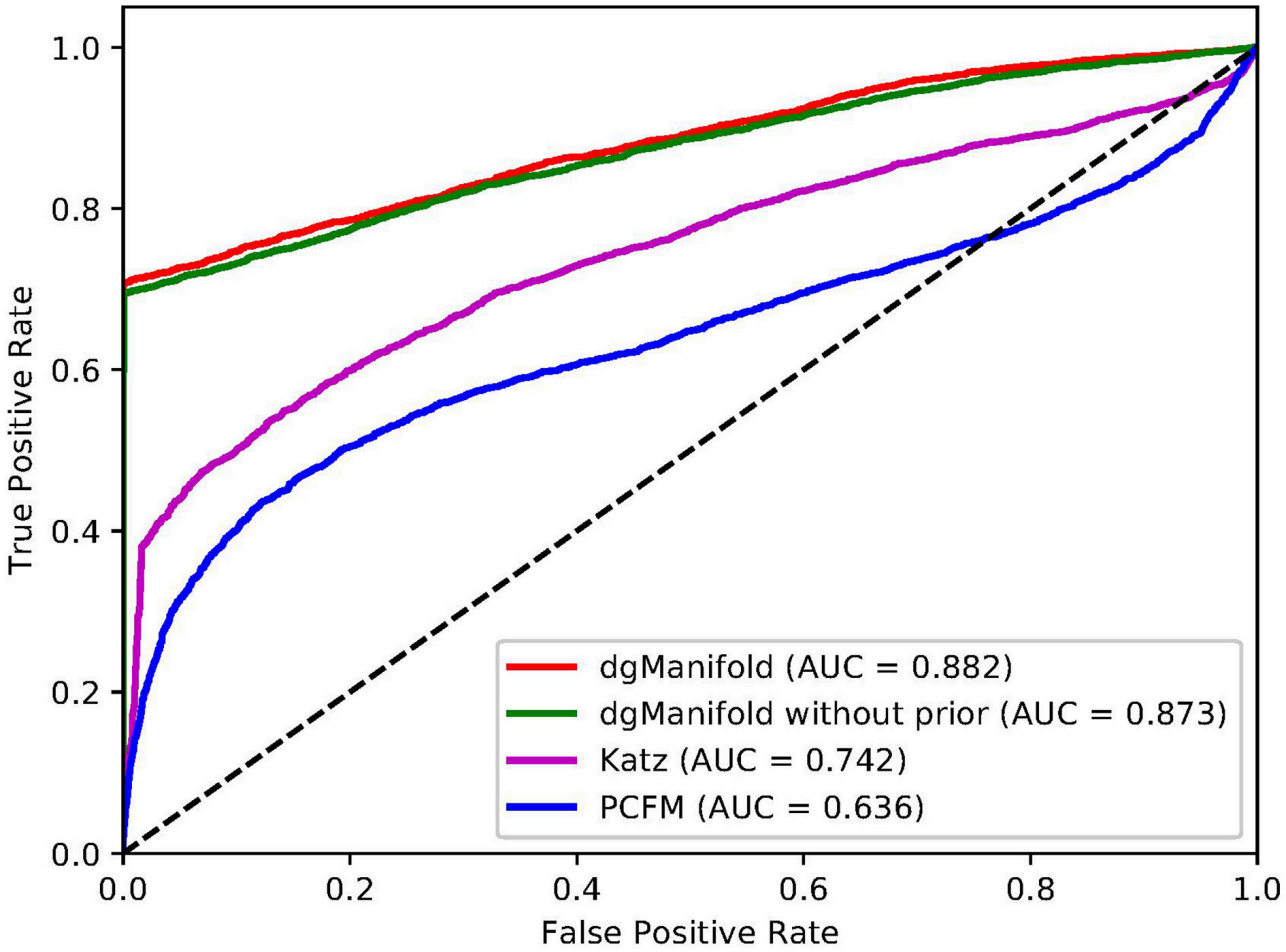
ROC curves of the three competing algorithms on multiple-gene diseases.

**Figure 2:**
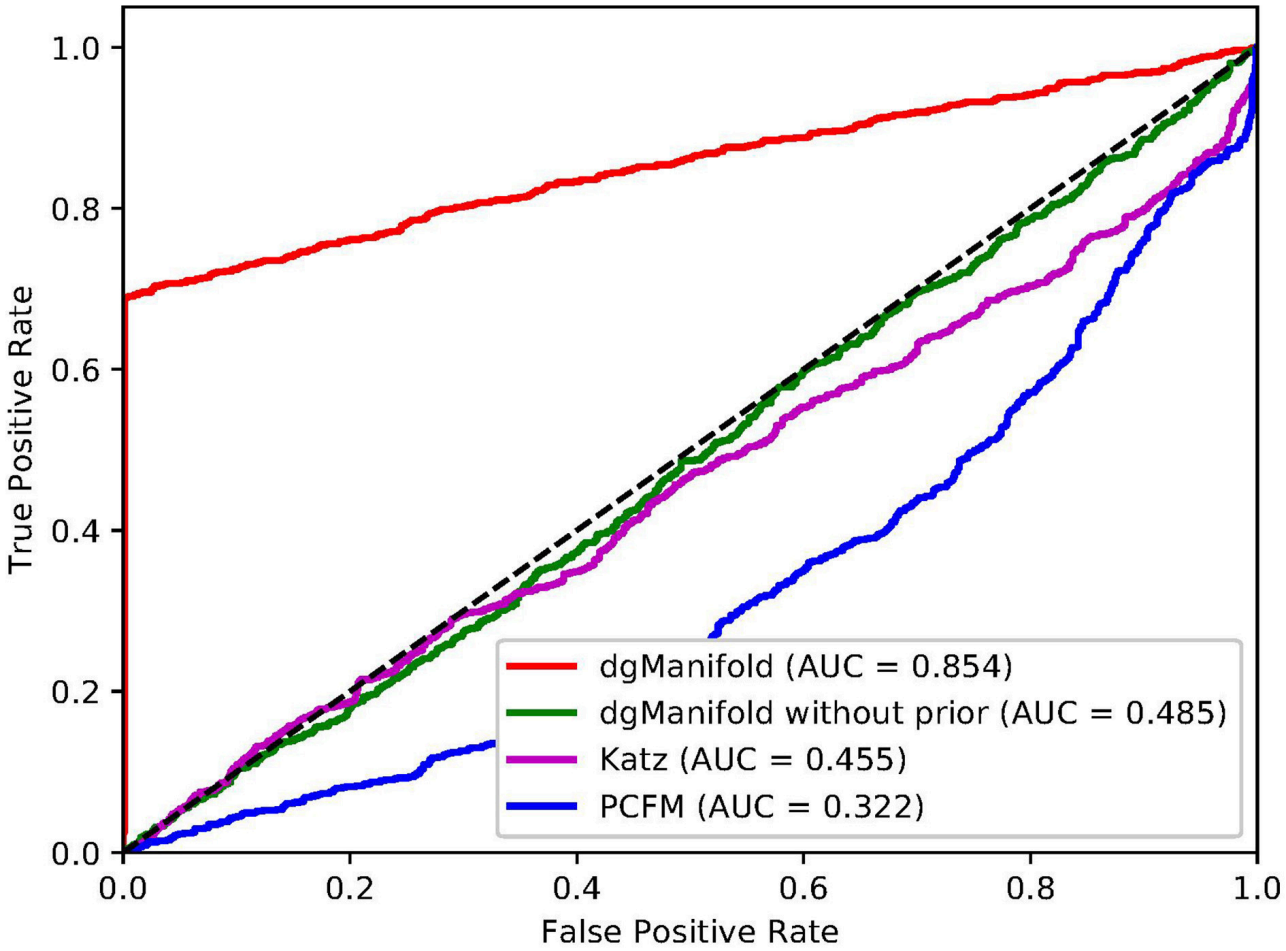
ROC curves of the three competing algorithms on single-gene diseases.

Figures 1, 2 show the resulted ROC curves and AUC scores of the three competing algorithms on multiple-gene diseases and single-gene diseases, respectively. For multiple-gene diseases, our dgManifold achieves AUC score of 0.882 with prior information and 0.873 without prior information, while the AUC scores of Katz and PCFM are 0.742 and 0.636, respectively. For single-gene diseases, the AUC score of our dgManifold is 0.854 when prior information is used and 0.485 with no prior information, while the AUC scores of Katz and PCFM are 0.455 and 0.322, respectively. These results show that our method is superior to the competing methods in terms of the AUC scores.

It is worth noting that the AUC scores of all three algorithms are less than 0.5 when they are applied to single-gene diseases. This is mainly because that single-gene diseases have no known associated genes during the cross-validation, and algorithms can only use disease similarities and association data of other diseases to perform the prediction. These data are not enough to generate accurate results, especially for supervised algorithms. Thus, prior information is necessary for the algorithm. In fact, the results of our experiments have shown that the prior information is beneficial to the prediction of disease-gene associations, especially when the diseases have no known associated genes.

### 3.3. *De novo* Study

In addition to AUC scores, we evaluate the performance of our dgManifold in predicting new disease-gene associations. Specifically, Lung Cancer and Bladder Cancer are selected, and prior information corresponded to these two diseases is added to matrix A. Then, all known disease-gene associations are used to train the model (*k* = 30, α = β = 0.2), and the geodesic distance between all the unknown disease-gene pairs is calculated. For each of the two selected diseases, the unknown disease-gene pairs are ranked based on the geodesic distance in ascending order, and the top-10 predictions are searched from existing literature.

Table 1 shows the results of *de novo* studies. 5 out of 10 predicted genes have been experimentally confirmed as associated with Lung Cancer. Among these genes, KCNK9 is a potential therapeutic target (Sun et al., 2016). HTRA1 contributes to the tumor formation by inhibiting the TGF-beta pathway (Esposito et al., 2006). ATP6AP1 and MYL2 are two potential biomarkers (Che et al., 2013; Sabrkhany et al., 2018). Mutation of C282Y allele in HFE is associated with Lung Cancer (McLarty et al., 2008). Although SEMA4A is not proved to be associated with Lung Cancer yet, it is related to Lung Inflammation and Colorectal Cancer, and its role in Lung Cancer genesis might be discovered in the future (Iyer and Chapoval, 2019). For Bladder Cancer, 3 out of 10 genes have been experimentally verified. Among them, SMAD3 mediates epithelial-mesenchymal transition which affects the invasion and migration of Bladder Cancer (Tong et al., 2018). DMP1 is a tumor suppressor gene of Bladder Cancer (Peng et al., 2015). CALR is potential biomarker (Kageyama et al., 2004). These results show that our predictions are in concert with existing reports, and thus our dgManifold is valuable for predicting new disease-gene associations.

**Table 1:** Top 10 predictions for lung cancer and bladder cancer.

**Gene symbol**	**References**
**LUNG CANCER**	
SEMA4A	
KCNK9	Sun et al., 2016
MYL2	Che et al., 2013
DENND5A	
HTRA1	Esposito et al., 2006
GABRA1	
ATP6AP1	Sabrkhany et al., 2018
KCTD17	
HFE	McLarty et al., 2008
BCS1L	
**BLADDER CANCER**	
PDYN	
DKC1	
SMAD3	Tong et al., 2018
MCC	
DMP1	Peng et al., 2015
MGP	
CALR	Kageyama et al., 2004
CASQ2	
SOX18	
GATM	

## 4. Conclusion

In this study, we have proposed dgManifold to predict disease-gene associations with manifold learning. Our dgManifold assumes that the distance between diseases and their associated genes should be shorter than that of other non-associated disease-gene pairs and maps the diseases and genes into a lower dimensional manifold based on known disease-gene associations, disease similarity and gene similarity. The prediction of new associations can be achieved by sorting the geodesic distance between unknown disease-gene pairs. The cross-validation results show that our model outperforms the competing algorithms in terms of AUC scores for both multiple-gene diseases and single-gene diseases. The further *de novo* studies also demonstrate that our dgManifold is valuable in predicting new disease-gene associations.

Note that dgManifold is only regularized by disease similarities and gene similarities at the current version, and the prior information is also obtained from the disease similarities. In the future, we can improve our method by regularizing the objective function with more types of data and computing the prior information with clinical evidences.

## Data Availability

The datasets generated for this study and a reference implementation of the algorithm can be found in the GitHub repository of the study.

## Author Contributions

F-XW conceived this study. F-XW, PL, QX, P-JW, and BL discussed about the methods. PL implemented the algorithm, designed and performed the experiments. PL and F-XW wrote the manuscript. All authors read and approved the final manuscript.

### Conflict of Interest Statement

The authors declare that the research was conducted in the absence of any commercial or financial relationships that could be construed as a potential conflict of interest. The reviewer MC declared a past co-authorship with one of the authors, BL, to the handling editor.
